# Digital Twin for Automatic Transportation in Industry 4.0

**DOI:** 10.3390/s21103344

**Published:** 2021-05-11

**Authors:** Alberto Martínez-Gutiérrez, Javier Díez-González, Rubén Ferrero-Guillén, Paula Verde, Rubén Álvarez, Hilde Perez

**Affiliations:** Department of Mechanical, Computer and Aerospace Engineering, Universidad de León, 24071 León, Spain; rferrg00@estudiantes.unileon.es (R.F.-G.); pverg@unileon.es (P.V.); ralvf@unileon.es (R.Á.); hilde.perez@unileon.es (H.P.)

**Keywords:** Digital Twin, AGV, Industry 4.0, simulation, smart manufacturing, cloud computing, hyperconnectivity, MIR100, ROS, Industrial Ethernet

## Abstract

Industry 4.0 is the fourth industrial revolution consisting of the digitalization of processes facilitating an incremental value chain. Smart Manufacturing (SM) is one of the branches of the Industry 4.0 regarding logistics, visual inspection of pieces, optimal organization of processes, machine sensorization, real-time data adquisition and treatment and virtualization of industrial activities. Among these tecniques, Digital Twin (DT) is attracting the research interest of the scientific community in the last few years due to the cost reduction through the simulation of the dynamic behaviour of the industrial plant predicting potential problems in the SM paradigm. In this paper, we propose a new DT design concept based on external service for the transportation of the Automatic Guided Vehicles (AGVs) which are being recently introduced for the Material Requirement Planning satisfaction in the collaborative industrial plant. We have performed real experimentation in two different scenarios through the definition of an Industrial Ethernet platform for the real validation of the DT results obtained. Results show the correlation between the virtual and real experiments carried out in the two scenarios defined in this paper with an accuracy of 97.95% and 98.82% in the total time of the missions analysed in the DT. Therefore, these results validate the model created for the AGV navigation, thus fulfilling the objectives of this paper.

## 1. Introduction

The industrial revolutions are characterised by technological advances that imply social and economic progress. Technological and scientific development in the recent years has enabled a new industrial revolution, the concept of Industry 4.0 [1]. For this reason, the paradigm of Industry 4.0 [2] proposes the digitalization of industrial processes with the ambition of increasing added value of products or services. Industry 4.0 aims the complete integration of all the agents involved (i.e., logistics, suppliers, financial services, trade) by implementing information technologies (i.e., TIC [3]). Therefore, connectivity is necessary to implement a collaborative technological network with the purpose of efficient and sustainable production.

Moreover, Industry 4.0 philosophy requires the implementation of recent technologies [4] (e.g., Internet of Things, Cloud computing, Digital Twin, AGVs). For instance, the implementation of the Internet of Things (IoT) provides the interconnection within the Industrial Ethernet Network (IEN) to create a collaborative environment that allows the interaction between machines (M2M) and services [5]. As a result of the IEN, the external services can be integrated into the industrial processes with the aim of increasing the optimization using smarts technologies. For this reason, Cloud computing [6] and Digital Twin (DT) [7] improves the efficiency and flexibility of the process planning in the manufacturing field. Parallel to the development of these services, a hyper-connectivity environment is being developed for facilitating the communication between industrial environment with other technologies [8]. Cyber-physical Systems (CPS) are smart devices that serve as a bridge between the physical and virtual worlds [9]. Moreover, these devices are equipped with connectivity to the IEN via IoT in order to send and receive data from other machines and services [10]. For this reason, Industry 4.0 allows the monitoring of the production processes dynamically and make smart decisions with the data of the industrial shopfloor.

In the last few years, few technologies associated with Industry 4.0 are implemented due to the incipient state of research [11]. Nonetheless, there are widely used technologies in the field of manufacturing (e.g., Radio Frequency Identification (RFID) [12], robots) that increase the quality and reduce the cost of products [13]. However, the lack of connectivity between machines and management services implies a reduction in the added-value of products [14]. In other words, the digitalization of machines is required to deploy CPS and is being currently developed to fully deploy the Industry 4.0 paradigm [15].

As a consequence of the globalization, technologies are implemented faster in the manufacturing industry in order to be competitive and to respond to market demands [16]. In this sense, the technologies used in the scientific literature aim to enhance manufacturing processes with different strategies (i.e., Lean manufacturing, Six-sigma) [17] without considering the coordination between machinery and services. A successful story is the implementation of these strategies in the 1980s by the Japanese industry in order to improve the productivity of the manufacturing industry [18]. Currently, companies have implemented Lean manufacturing, however, a collaborative and connected environment is required in all manufacturing processes in order to increase productivity.

Furthermore, responding to the increase of customized manufacturing, the industrial requires making manufacturing processes more flexible [19]. Therefore, Industry 4.0 increases flexibility on the shopfloor by automating the industrial organization through the use of hyperconnectivity-based techniques that facilitate the transfer of information. For instance, artificial intelligence-based algorithms (i.e., Job Shop Scheduling Problem (JSSP) [20], Facility Layout Problem (FLP) [21]) can be applied to solve problems automatically, thus achiving a better decision-making.

Due to the increasing development and implementation of these concepts, new terms (e.g., Smart Manufacturing (SM), Computer-Integrated Manufacturing (CIM)) have emerged with the aim of adapting to the rapid changes in the manufacturing industry. SM [22] complements the Industry 4.0 paradigm with the use of specific technologies (e.g., predictive engineering, IT platforms, machine vision, collaborative robots, automation of internal logistics) which allow an increase of value in the production chain [23].

Historically, there have been various technologies for automating internal transport in industry with land vehicles in order to reduce the costs associated with this problem [24]. Nowadays, in the context of SM, the automation of transport in the manufacturing plant is a new challenge in the scientific literature due to the emergence of disruptive technologies (i.e., computing, Artificial Intelligence (AI), smart sensors, battery autonomy, wireless connectivity). By means of embedded intelligence in Automated Guided Vehicles (AGVs), autonomous navigation is achieved allowing the obtention of greater operability in the manufacturing plant. In addition, the connectivity currently incorporated in AGVs allows the integration into a collaborative environment, optimizing fleet routes in real-time while coordinating with other machines [25]. In general, AGVs reduce the cost and time of internal industrial logistics, achieving greater productivity, which means being more competitive.

The deployment of AGVs in the manufacturing plant brings more advantages than traditional transport systems. Furthermore, real-time connectivity between management and AGVs permits communication without human analysis errors [26]. Due to this connectivity of the AGV fleet, the optimization of trajectories and the traceability of goods is improved. In the field of occupational safety, the accident of AGVs (e.g., run-overs, loss of goods) is much lower than that of conventional transport. Furthermore, the new technologies incorporated in AGVs permit greater flexibility, allowing them to adapt in real-time to new situations without the need for human intervention. Therefore, AGVs complement the new paradigms of Industry 4.0 and SM.

However, the implementation of AGVs in the industrial environment presents a series of challenges that the scientific community is nowadays addressing. One of them is the improvement of positioning techniques in indoor environments [27], as it is critical to know the exact location for the calculation of trajectories [28]. Another problem is to meet the demanding wireless connectivity characteristics (i.e., latency, bandwidth, coverage) of AGVs, and to improve the route optimization algorithms in order to meet the real-time demands of the workstations. Another line of research in this field is the creation of DT to analyse the behavior virtually and allowing the identification of potential problems in the actual deployment of AGVs thus supposing a cost reduction in the implementation of these vehicles in the manufacturing plant.

In the context of DT, the scientific literature proposes modelling AGVs to predict their behaviour in different scenarios [29] but it has not yet been addressed in a real-case study. In this way, manufacturing processes can be virtualised by analysing several parameters of both the AGV and the environment. Furthermore, by using DT, the costs associated with testing in the real world are reduced since a software-based process virtualisation is more cost-effective [30]. Furthermore, an improvement in the process planning is obtained by reducing uncertainties.

The DT currently available in the scientific literature do not model the environment or the behaviour of AGVs. Current simulations consider basic parameters (e.g., route distance, speed, acceleration) [29] while ignoring all elements of the environment involved in the autonomous navigation of AGVs [31]. Consequently, the dynamic response of the AGV to the virtual environment is not taken into account. Consequently, processing times in real environments differ from the current simulations [32].

For this reason, we propose the creation of a platform that allows the complete virtualisation of the scenario as well as the AGV modelling. For addressing this purpose, we introduce the first model of the trajectories and navigation of an AGV in the industrial plant which allows the replication of actual conditions of operation enabling the simulation of the behaviour of the AGV in order to help decision taking in the Industry 4.0 paradigm.

Furthermore, the scenario can be modified at runtime, increasing the dynamism and realism of the simulations. All actions on the AGV are automated by means of a Human Simulation Interface (HSI). At the same time, we provide the DT with hyperconnectivity [8] to external services (e.g., e-mail, comma-separated values (CSV), Telegram, Social networks) for the automatic export of data using reports.

In order to implement these concepts, an IEN is required, which is managed by a router equipped with a firewall [33] to provide cybersecurity. For this reason, we propose in this paper a communications architecture to integrate external services as well as the elements of the manufacturing plant in order to create a collaborative environment. On the other hand, for the implementation of the DT, the Robot Operating System (ROS) [34] environment has been used for the programming of the autonomous navigation of the AGV [35]. Furthermore, the designs have been incorporated with real scenarios for virtualisation in order to be able to compare the results with real devices. The results obtained in this paper show the validity of the DT proposed and suppose an achievement in the SM paradigm through the proposal of the first DT for AGVs navigation with actual proved effect in the scheduling of the Material Requirement Planning (MRP) [36] in the industrial plant in the authors’ best knowledge.

The remainder of this paper is organised as follows: Section 2 describes and introduces the concept of the DT in the context of the Industry 4.0 in order to identify novel applications of this technology in the industrial environment. Then, Section 3 explains the objectives of the implementation. Section 4 describes the developed communications architecture. Subsequently, Section 5 analyses the external service of the DT in detail by defining the applications that are used in the DT. Then, in Section 6, the simulation scenarios are described and the results are presented in Section 7. Finally, the paper is concluded in Section 8.

## 2. Digital Twin from Industry 4.0 Conception

In the context of Industry 4.0, DTs are defined as the virtualisation of physical systems for predictive analytics [37] considering the replication of the CPS in the virtual world [38]. However, the DT concept appeared for the first time in 2005 by Grieves in order to manage the product lifecycle [39]. Later, in 2012, NASA used the DT for the first time as a virtual model of a physical system [40]. Currently, DTs are used in industry in order to optimise the modelling and analysis of product properties prior to manufacturing, which increases the value of the product. Various types of DTs (e.g., product DTs, production DTs, performance DTs) [41] are described in the literature with the aim of creating a framework of DTs that connects all stages of the product lifecycle with MES [42].

DTs are also considered as a virtualisation of an environment to analyse its performance in hypothetical situations [38]. The applications (e.g., design, production, full lifecycle) have in common the analysis of the response of the systems [43]. However, in other papers they are applied for production optimisation by simulating the processes [44]. In this paper we propose for the first time in the authors’ best knowledge the integration of DTs with industrial planning and management systems (e.g., MES, ERP).

Furthermore, the MES enable production to be controlled and monitored so as to achieve maximum efficiencies and cost reductions. Therefore, the MES are critical for optimising production planning in the era of Industry 4.0, which is characterised by digitalisation and an increased integration of processes throughout the supply chain [45].

For this reason, The use of DTs in process planning would provide high-value information to the MES in new processes and achieve more realistic planning. In order for the DTs to convert data into information, it is necessary to define a process transfer function (i.e., in the modelling of the CPS [46]).

**Figure 1:**
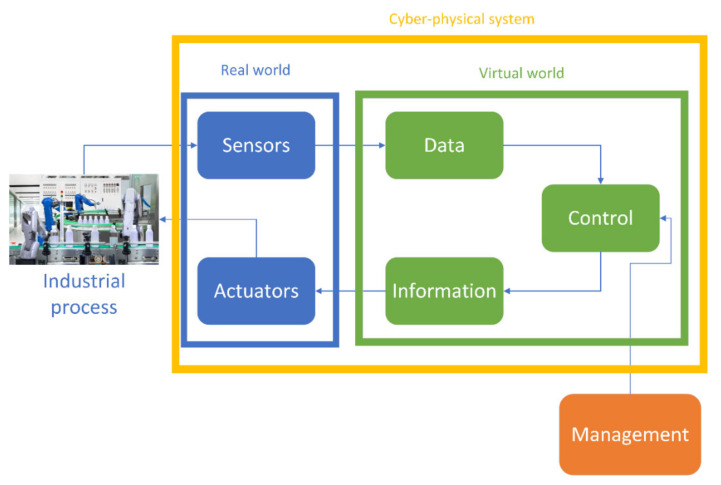
Structure of elements constituting cyber-physical systems.

In the context of Industry 4.0, the CPS are defined as a devices equipped with intelligence and connectivity that enables the link between the virtual and the physical worlds [4]. On the one hand, the virtual world provided by TIC allows the processing and visualisation of data (i.e., Control, Supervisory Control And Data Acquisition (SCADA), BigData [47]) and also the communication among its applications [48]. On the other hand, the real world captures data provided by sensors, actuators and machines installed in the industrial plant, enabling the modifications and perceptions of the physical variables of the manufacturing processes. Therefore, CPS convert physical units into data and information into actions, as can be seen in Figure 1.

Hence, in order to predict the CPS actions, the DTs virtually replicate physical world assumptions in order to interconnect both worlds. Therefore, CPS modelling is essential to accurately virtualise the operations of the physical world. Not only do DTs virtualise processes, but also generate high-value data for the MES, where data are analysed to assess feasibility and product quality.

Consequently, the virtual models reduce the necessity of testing in the physical world with its associated costs. However, to ensure continuous improvement, some DTs are defined as a virtual model with the need of feedback of physical processed data [49]. Even so, correct modelling of the CPS and a dynamic virtualisation of the environment yield good results without the necessity for real data beforehand.

The analysis of the deviations between the results obtained and the real ones will allow for continuous improvement [49]. The fact of obtaining results without the need to have real data beforehand is a considerable advancement in the planning of new industrial processes in this paper.

The development of automated transport in the industrial plant with AGVs has brought new challenges for production planning [24]. One of these challenges is the integration of transport with production processes in order to fulfil continuous and dynamic demands [50]. For this reason, the analysis of transport times is essential to establish an efficient planning and to guarantee the supply of goods and the material requirement satisfaction in the industrial plant [51].

Due to the complexity of the navigation systems and the dynamic evolution of the manufacturing plant, it is difficult to determine accurate travel times. In this problem, DTs can be applied considering the AGV as a CPS [52], as well as a scenario allowing the virtualisation of the CPS environment.

The integration of DT with other technologies of Industry 4.0 (e.g., IoT, AI, Augmented Reality (AR) [53], Virtual Reality (VR)) is not yet be fully developed. For this reason, in this paper, we propose the creation of a new framework for DTs to integrate different Industry 4.0 and SM technologies for the first time in the authors’ best knowledge.

Due to the use of new technologies, AGV routes have been automated by creating customized missions [54]. In the context of DTs, simulation interfaces are not sufficiently discussed in the current literature. For this reason, we integrate hyperconnectivity systems to improve human-simulation interaction. Furthermore, by applying these techniques, the generation of simulation reports and their integration with MES can be automated.

Furthermore, in the current scientific literature, DTs do not dynamically modify the environment, so virtualisations of specific processes (e.g., autonomous navigation) have not been investigated. For this reason, the development of this framework permits more accurate results to be obtained by virtualising the scenarios [55]. Furthermore, the techniques employed in this paper do not require previous real data since the modelling of the environment and the AGVs is complete. However, it is necessary to define the real scenario in order to virtualise the simulation.

Consquently, a real scenario and vehicle data are required for the full virtualisation of the transport in the industrial plant. A comparison of the results in both the real and virtual worlds is made to validate the methodology implemented.

## 3. Objectives and Methodology

In this paper, a real implementation of the AGV transportation in the industrial plant is needed to validate the proposed objectives detailed hereafter:

- Definition and design of a communications architecture to provide connectivity to the equipment located in the industrial plant which constitutes the IEN.
- Access the IEN from the outside in order to monitor and control processes allowing an external intelligent process planning.
- Implement cybersecurity standards such as ISO/IEC 27001 to protect the industrial plant from any internal or external threat. Therefore, the security of IEN equipment and information is increased.
- Provide connectivity to the new service associated with the DT by integrating it into the IEN. In this way a Modelling and Simulations as a Service (MSaaS) is developed [56].
- Definition, design and programming of the framework associated with the DT service.
- The virtualization of the real scenarios in the virtual environment with the aim of comparing both results.
- Modelling of the AGV to predict its behaviour in virtual environments.
- Design and programming of the HSI where the simulation will be monitored. In the HSI, data is visualised and actions are transmitted.
- Automate actions in the simulation for the elaboration of user-customised missions.
- Extracting information associated with the simulation results in different formats and media using hyperconnectivity.

All these objectives are implemented both in the real and virtual worlds through the use of the communications architecture detailed Section 4 and through the DT framework introduced in Section 5.

The methodology used for the experimentation of the DT is based on the virtualisation of the environment and the modelling of the AGV. The virtualisation has been implemented in two real scenarios to recreate them in the DT. In addition, the modelling of the AGV has been carried out according to its technical properties and with the same configuration as that of the DT. Therefore, a series of objectives are established by simulating the work in an industrial plant both in the DT and in the real scenarios. The data extracted from the DT and the real AGV have been compared to evaluate the accuracy of the DT.

## 4. Communications Architecture

In the context of Industry 4.0, a new concept of communications is established due to the new requirements of industry. In this regard, the widespread digitisation of processes with CPS, flexibility and greater complexity in automation requires this change in the connectivity of both industrial equipment and the services provided. Due to the development of TIC together with the improvement of the internet network, the implementation of the Industrial Internet of Things (IIoT) [57] improves connectivity in industry. In addition, the 5G mobile network provides the industry with wireless connectivity with reliability and quality standards allowing an improvement in the Quality of Service (QoS).

Furthermore, manufacturing processes are more complex and require more advanced techniques (e.g., machine vision) which implies higher bandwidth. Due to the implementation of new techniques in industry, the fieldbuses used as well as the protocols did not meet the needs. Therefore, industrial networks based on the IEEE 802.3 [58] standard protocols were developed, thus unifying the criteria for Ethernet networks. In this way the IEEE 802.3 standard defines the physical and operate in a link layer which were later added to the OSI model [59]. For instance, networks based on Industrial Ethernet support different protocols (e.g., Profinet, OPC UA, EtherCAT, Modbus TCP) and physical media (e.g., coaxial cable, fiber optics, twisted pair). The physical media of Industrial Ethernet are prepared for hostile environments (e.g., electrical noise, vibration, extreme temperatures), as reliable communications are essential. However, half-duplex networks need protocols to guarantee the quality (e.g., Carrier Sense Multiple Access with Collision Detection (CSMA/CD) [60], Acknowledgement (ACK)) of communications.

The earliest Ethernet networks in industry were used for management and planning where TIC technologies are used. Over time, manufacturers of industrial instrumentation have opted for Ethernet networks as a method of communication between control systems (e.g., (PLC), (Distributed Control System (DCS)) and monitoring systems (e.g., HMI, SCADA). Industrial communications based on Industrial Ethernet (IE) [61] are therefore the present in the field of research.

**Figure 2:**
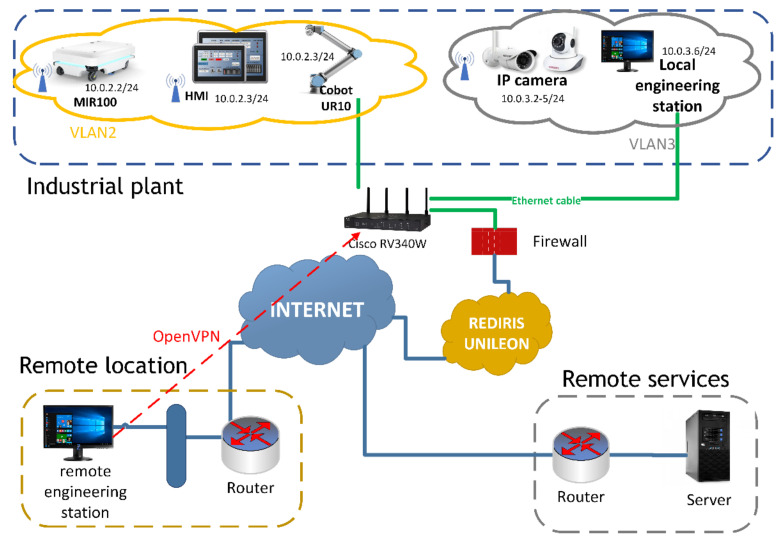
Proposed communications architecture for the Industry 4.0 and SM demonstrator.

In this paper, we create an architecture in Figure 2 based on IE in order to demonstrate the connection between the different technologies involved in Industry 4.0. In addition, a communication with the outside of the industrial plant will be provided in order to outsource the control and monitoring. For this purpose, cybersecurity criteria are established to guarantee the integrity of the information and equipment.

As it can be observed in Figure 2, the communications architecture is divided into three sections; the industrial plant, the external service and the remote engineering station.

- The industrial plant is modelled in the E3 laboratory and the hall of the engineering school of the University of León. This is where some of the equipment (e.g., MIR100, UR5e, cameras, engineering station) necessary to simulate and develop industrial processes related to transport is located.
- The external service consists of an internet-connected server from which the industrial plant and the remote engineering station can be accessed. This external service is used to perform computational tasks necessary for process optimisation. This avoids installing computers in the plant with its associated problems (e.g., dust, electrical noise, ATEX zone [62]). By outsourcing the DT’s computer service, no maintenance is required at the industrial plant and the software remains privately owned. The service can be accessed remotely from the industrial plant using the network infrastructure, such as cloud computing. In this paper, the DT service runs from this external service.
- The remote location of the engineering station makes it possible to delocalise the supervision of the industrial plant from any device connected to the internet. In addition, external services can be accessed from the several device if preferred.

### 4.1. Industrial Ethernet Network (IEN)

The IEN provides routes between the equipment located in the industrial plant by means of a router. This router divides the IEN from the network provided by the redIris IPS. For this purpose, the physical medium used in the fixed equipment is twisted pair cat6 cable, allowing a bandwidth of up to 10 Gbit/s. However, for mobile equipment (e.g., AGVs), the connection is made via the IEEE 802.11 standard or WiFi, creating a Wireless Local Area Network (WLAN) [61]. The channels used for WLAN connections are the 5 GHz band, which allows a maximum speed of 1.3 GBit/s at a reduced range. However, the 2.4 GHz band has a longer range but its maximum speed is 450 MBit/s.

In this sense, secure access is provided by Virtual Network Protocol (VPN) [63] which allows the connection from the external device to the IEN. The VPNs may be configured to allow access to a single device (i.e., client to client) or to the entire IEN (i.e., client to site). In industry, both are used depending on the role of the user. The administrators have access to the network while the operators or manufacturers only have access to the established device.

### 4.2. Outside Connection

One of the innovations of this architecture with respect to existing ones in the scientific literature consists in providing connectivity with the outside world. In this case, connectivity is offered by the Internet Service Provider (ISP) RedIris, which provides a static Internet Protocol version 4 (IPv4) address. This address is redirected by using the Domain Name System (DNS) that provides the communications service of the University of León, which redirects the name to the public IP address in order to facilitate access.

Furthermore, the implementation of connectivity with the outside of the industrial plant allows the implementation of hypercontivity. For this purpose, the engineering station includes a server that acts as a gateway. This server integrates the industrial communications protocols with the APIs of third-party services (e.g., cloud computing, VR or AR services, IoT devices). In addition, simple algorithms can be implemented in the gateway to reduce latency and improve the QoS.

### 4.3. Cybersecurity

The connection of a private industrial network to the internet implies a multitude of risks (e.g., access to equipment configuration, access to data, process control) and it is critical to minimise these risks. Therefore, separating the IEN from the Wide Area Network (WAN) (i.e., RedIRIS) by implementing a firewall that blocks unauthorised access is necessary in order to guarantee security. In addition, the firewall protects against service blocking attacks (i.e., Distributed Denial of Service (DDoS) [64]). Additionally, the firewall protects against suspicious packets by performing a Deep Package Inspection (DPI) [65] or by content filtering. Besides all of the above, both the router and the firewall have defined a table establishing which ports can be accessed and which devices.

Furthermore, most cybersecurity breaches occur within the network are due to user negligence. For this reason, inside the IEN, the network has been segmented using Virtual Local Area Networks (VLANs) [66]. As a result, the user of one VLAN cannot connect to another VLAN and thus improving the cybersecurity. In addition, devices can communicate with each other due to the definition of Inter-VLAN Routing rules. As it can be seen in Figure 2, the architecture presented in the industrial plant it has been segmented according to the equipment used in order to prevent some users from accessing restricted equipment.

## 5. Digital Twin as an External Service

In this paper, we propose a new concept of DT offered as a remote service. In this way, we extend MSaaS applications [56] by making use of the proposed communication architecture and Cloud Computing [67]. This innovative concept improves connectivity in industry and can be applied to other problems (e.g., JSSP, FLP). However, in this paper, we focus on the problem of automated transport in the industrial plant with AGVs. Subsequently, the results of the simulations will be compared in reality to evaluate the performance of the DT.

To this end, a new framework designed for remote programming and use is proposed. In addition, the first HSI in the scientific literature is implemented for the DT with the aim of facilitating the integration between the human and the virtual world. Moreover, the HSI users will be able to access from any device to manage and monitor the DT simulations.

### 5.1. Framework

The external service of the DT is running on a remote serve. Regarding communications, the server is connected to the internet with a dynamic IPv4 using a different ISP than the University of León, so it is an external and independent server. For access, a dynamic DNS service is used which modifies the public IPv4 to the assigned domain name. In addition, the server uses an Ubuntu operating system version 20.04 which supports the installation of the different libraries and applications used in the DT.

**Figure 3:**
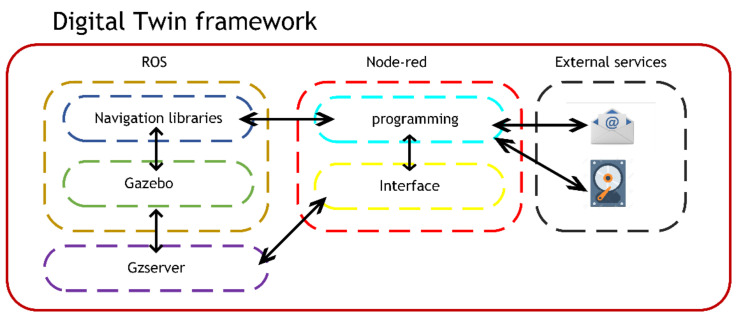
Framework of applications and services for the development of of the DT proposed for the automatic transportation in the industrial plant.

The framework has been designed to execute the AGVs modelling and virtualisation scenarios that are required for the DT. Furthermore, it integrates new technologies (i.e., hyperconnectivity, IIoT, cybersecurity) and concepts associated with Industry 4.0 and SM. For this purpose, the networking of different applications and libraries that have never before been interconnected in the scientific literature has been required. Besides, different communication protocols have been used to achieve the implementation of the DT remote service. An schema of the different applications is shown in Figure 3.

As shown in Figure 3, the framework of applications and libraries is structured in three sections; the ROS ecosystem, the Node-red server and third-party services.

- The ROS ecosystem is composed of the navigation libraries and Gazebo [68]. The navigation libraries communicate bi-directionally with the Node-red server program and the gazebo application. The Gzserver is a server that runs in parallel to the Gazebo application allowing access to the application from a web browser.
- The Node-red programming environment communicates bidirectionally with external services (e.g., e-mail, database) conferring hyperconnectivity to the DT. Furthermore, the Gzserver application has been embedded in the HSI to monitor the simulation in real-time from a single interface.
- From external services and applications (e.g., MES, SCADA) information can be sent to run simulations. Besides, this server can be used as a gateway to provide hyperconnectivity to other applications with cybersecurity in mind.

### 5.2. ROS Ecosystem

ROS is an open-source framework with several toolkits to solve robotics-related problems. Its operation is based on nodes, which communicate using the publisher-subscriber philosophy. For this reason, each node is able to send and receive information from other nodes. Furthermore, nodes are able to run programs for device control, device status, among others. Because of the existence of several nodes, the information processing is distributed, although all the nodes are running on the same device.

Communications are carried out between nodes through the use of topics. Every topic is a unique and independent channel where nodes can publish (i.e., send messages) and subscribe (i.e., receive messages). In this way a node (i.e., navigation library, gazebo, sensors, actuators) can publish and subscribe to multiple topics. This publisher-subscriber philosophy is utilised in other communication protocols such as Message Queuing Telemetry Transport (MQTT) [69]. However, in ROS, messages are structured in JavaScript Object Notation (JSON) in order to be able to send information from one node to another regardless of the programming language used in each node.

Furthermore, ROS classifies the types of messages and establishes their structure. In this way, the information transmitted from a sensor (e.g., Laser Imaging Detection and Ranging (LIDAR), ultrasound onboard of the AGV) is structured in the same way regardless of the model used. As a result of this feature, the flexibility and compatibility of the robotic models used with the navigation libraries is improved, which allows the reuse of the code. However, successful communication requires the completion of all the fields set out in that message type.

Besides, ROS can also communicate with third-party applications by programming bridge nodes using the WebSocket protocol [70]. This feature provides connectivity and coordination with other independent devices in the ROS ecosystem. In this paper, we have opted to externalise the automation of missions outside the ROS framework to another server in order to provide more connectivity.

**Figure 4:**
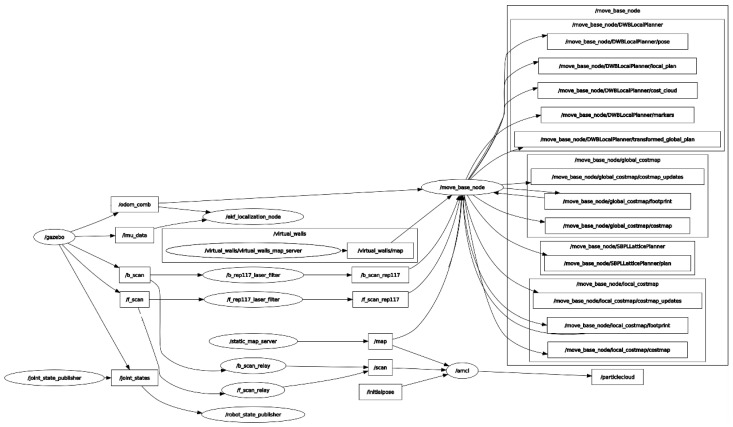
Diagram of communications among nodes of ROS ecosystem.

The Figure 4 shows a diagram of the nodes and topics used in the development of the ROS ecosystem.

In Figure 4, the ellipses correspond to the nodes, while the rectangles identify the topics. In this diagram, some nodes publish information to which nobody subscribes, which means that this information is not yet received by any other node. The arrows indicate the direction of the messages. For instance, inbound arrows to the node imply that this node is subscribed to that topic. However, outgoing arrows to the node mean that the node is publishing to that topic. The most important nodes are “*move_base_node*”, “*amcl*”, “*Gazebo*” and “*Node-red*”, the rest of the nodes correspond to sensors and actuators of the AGV.

- *Move_base_node* aims to model the AGV behaviour. In order to model the movement of the AGV, a navigation stack is required to recreate the movements of the real model. For this purpose, the node subscribes to the information offered by the sensors that the AGV incorporates as well as the navigation goals in order to publish the response to the actuators. In addition, this node publishes information in simulation time such as the state of the AGV, the position, the linear and angular velocity, among other parameters. Furthermore, for a correct simulation, it is necessary to know where the actuators (e.g., wheels) and sensors (e.g., LIDAR) are located in order to model the dynamic behaviour of the AGV. For this purpose, the parameters are defined in a Unified Robot Description Format (URDF) [71] file in XML format which represents the model of the AGV.
- The virtualisation of the scenario which is necessary for the development of the DT is performed through the programming of Gazebo. This node defines the space where the AGV will navigates with the definition of walls, objects and obstacles. Under this same scenario, the three-dimensional model of the *move_base_node* will be loaded in order to simulate the information received by the sensors (e.g., LIDAR, ultrasound) that depend on the environment. In this way it is possible to simulate the data that would be obtained in a real scenario. These data are published to the *move_base_node* that elaborates a response that will receive the virtual model in Gazebo. In the simulated environment, the AGV will move accordingly, generating new data and completing the loop. Furthermore, from this node it is possible to import three-dimensional models that have already been designed in order to provide the DT with greater realism. In real-time simulation, the virtual environment can be modified by observing the dynamic response of the AGV. In addition, the environment can be altered in simulation time in the same way as it happens in reality.
- The Node-red is used as a bridge to communicate with the Node-red server responsible for the supervision and control of the virtual simulation. This node is subscribed to the information (e.g., position, navigation status) of the *move_base_node*. Besides, this node publishes the navigation goals due to the automation of the missions by Node-red.
- The Amcl node is responsible for calculating the position of the AGV in the environment. For this purpose, it uses the LIDAR data in conjunction with the previous mapping to calculate the position. Based on the position information, it is important for decision making in the autonomous navigation of the AGV.

### 5.3. Node-Red Server

Node-red is a platform that enables the connection of various online services and APIs to the IoT. Node-red is a flow-based editor over a web browser. The programming language of this server is JavaScript with a Node.Js environment, which is ideal lightweight and robust, making it ideal for industrial applications. In addition, the interface, as well as the programming environment, has a web interface which allows it to be multiplatform. Furthermore, it can be accessed from the internet after configuring the network in order to guarantee cybersecurity. In this sense, multi-client access is possible, and other users’ modifications can be seen in real-time.

In the case of the server implementation, the execution of Node-red runs on the same hardware as the ROS ecosystem. Therefore, latencies are reduced, improving the response between ROS and Node-red.

Furthermore, part of the objectives of programming with Node-red is the automation of the ROS ecosystem missions. For this purpose, a bridge must be established between the nodes of the ROS ecosystem and Node-red. Therefore, the Node-red node of the ROS ecosystem can subscribe and publish on any topic. In this way, the information is obtained and transmitted between Node-red and the ROS ecosystem.

**Figure 5:**
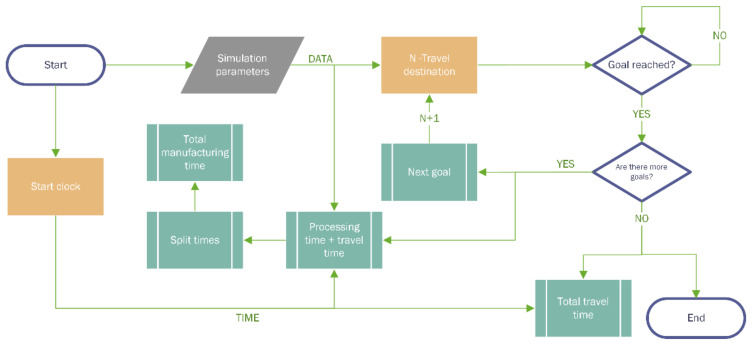
Code flowchart for the automation of actions in the Digital Twin.

With established communication, the actions can be automated by scheduling events. In this sense, accurate control of the simulation can be attained due to the access of all information from the ROS ecosystem nodes in real-time (e.g., AGV position, targets reached, navigation status). In Figure 5, the scheduling of the missions with a pseudo-code is detailed.

In addition, this server offers a DT interface for ease of use, without the need for programming. Moreover, if the automation needs are modified, only programming is required on this server without the need to modify the code of the ROS ecosystem. All of these considerations suppose a novelty in the current scientific literature.

Furthermore, Node-red facilitates connectivity with other third-party applications. Therefore, it is possible to export data in different protocols, thus achieving the concept of hyperconnectivity. This facilitates communication among applications, reducing human errors as well as costs.

## 6. Scenario Description

In order to verify the theoretical concepts, a demonstrator is implemented applying all the technologies associated with Industry 4.0 and SM. For this purpose, the architecture mentioned in Section 4 is developed in the real world, which provides connectivity to the industrial plant and to the remote services. Therefore, the remote DT service on AGVs detailed in Section 5 allows the simulation of the AGV behaviour in a virtual environment. For this reason, the modelling of the real environment is critical to obtain comparable results with the virtual AGV. As a consequence, the scenarios implemented in the virtual world are the same as in the physical scenarios where the real-world demonstrations are performed. Hence, the real and virtual behaviour are comparable in order to validate the DT model.

The demonstrations are carried out in two different scenarios. *Scenario 1* recreates the behaviour of the AGV in confined spaces. *Scenario 2* is a larger space to evaluate the response of the AGV. In this sense, the aim is to compare the behaviour of the trajectories as well as the time spent in the trips carried out by the virtual and real AGV.

### 6.1. Targets and Missions

The demonstration consists of simulating the transport of materials in the industrial plant in both the real and virtual world. The targets are defined corresponding to processes associated with the machining of manufacturing pieces (i.e., drilling, turning, milling). For this purpose, the AGV travels to the four targets established in each scenario in order to measure the duration of all the journeys. Therefore, each scenario has a single start and end point, while the other three positions of the manufacturing machines are distributed around the industrial plant. In addition, the orientation of each target has been considered for the navigation of the AGV in the missions. The orientation influences the route planning because the navigation space is defined by coordinates on the *x*-axis and *y*-axis and an angle which represents the orientation.

The missions are composed of four trips, with the start and end locations being the same for all missions. Hence six possible combinations result from changing the order for the three processes are six. For this reason, each scenario has six different missions which will be studied in order to compare the results of the real and virtual world. The details of each scenario are defined hereafter.

### 6.2. Scenario 1

**Figure 6:**
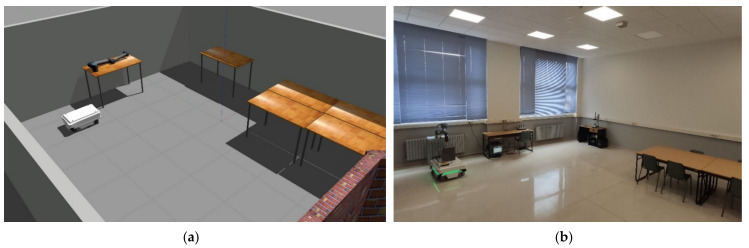
Both figures show Scenario 1 (**a**) It represents the virtualisation of the AGV by the DT service; (**b**) The E3 laboratory of the engineering school of León where the experimental tests are carried out.

Scenario 1 is located in the E3 laboratory of the School of Engineering of the University of León. This rectangular open-plan scenario has an area of 71 m^2^. The purpose of this space is the recreation of a workshop or work cells in industry. For a better understanding of scenario 1, a comparison between the real and the virtual scenario is presented in Figure 6.

The Cartesian coordinates adopted are based on the lower left corner of the industrial plant. In terms of orientation, the zero-degree reference is placed on the *x*-axis moving counterclockwise. The following Table 1 shows the coordinates and orientation assigned to each process.

### 6.3. Scenario 2

**Figure 7:**
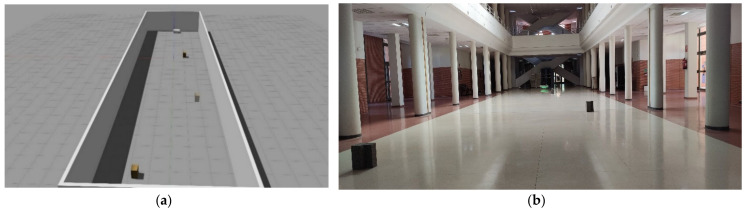
Both figures show Scenario 2 (**a**) It represents the virtualisation of the AGV by the DT service; (**b**) The hall of the engineering school of León where the experimental tests are carried out.

Scenario 2 is located in the hall of the engineering school of the University of León. This rectangular scenario has a side of 30 m and another of 7 m, so the total area is 210 m^2^. The navigation zone of the AGV has no permanent obstacles (e.g., columns), so it is an open space. This area is defined in the real scenario by the white colour of the floor while in the virtual scenario it is represented by walls since the AGV navigation requires these references for the appropriate navigation of the automatic vehicle. However, in the mapping of Scenario 2 by the real AGV shown in Figure 7, virtual walls have been programmed so that the environments are the same in order to evaluate the results.

The Cartesian coordinates used have their origin on the lower-left corner of the industrial plant. In terms of orientation, the zero-degree reference is placed on the *x*-axis moving counter-clockwise. Below in Table 2 the coordinates and orientation assigned to each process are set out.

## 7. Results

Industry 4.0 and SM technologies have been used to implement a demonstrator in order to verify the theoretical concepts. In this sense, the communications architecture based on the Industrial Ethernet of Section 4 provides communication with all the devices and equipment used in the real world. Furthermore, the communication architecture allows communication with the DT service. Therefore, the DT explained in Section 5 simulates the behaviour of the AGV in specific environments. For this reason, two scenarios, detailed in Section 6 with different characteristics, are used. For the purpose of validating the DT, it is required to compare the results of the virtual world with the real implementation.

An analysis of the times associated with the transport of AGVs is the most appropriate way to compare the DT with the actual AGV. In this respect, the time is a parameter that influences the scheduling of various nodes (i.e., environment analysis, navigation routes). Moreover, a time correlation between the real world and DT would validate its use for MES systems in order to improve transport planning in the industrial plant. For this purpose, different environments are analysed, which are presented below.

### 7.1. Characterization of the Mission Parameters

In this paper an implementation of the theoretical proposal is carried out in order to validate it. Moreover, in the field of DTs, it is required to consider a multitude of parameters both in the physical world and in the virtual world in order to obtain comparable and reproducible results. The experiments have been carried out under the following hypotheses in the Table 3.

### 7.2. Scenario 1

In Scenario 1 the AGV travel times indicated in Table 4 are lower due to shorter distances. However, high speeds are not achieved in this Scenario due to the proximity of walls and targets. In addiction, the accuracy between DT times and the experimental tests is very high.

**Figure 8:**
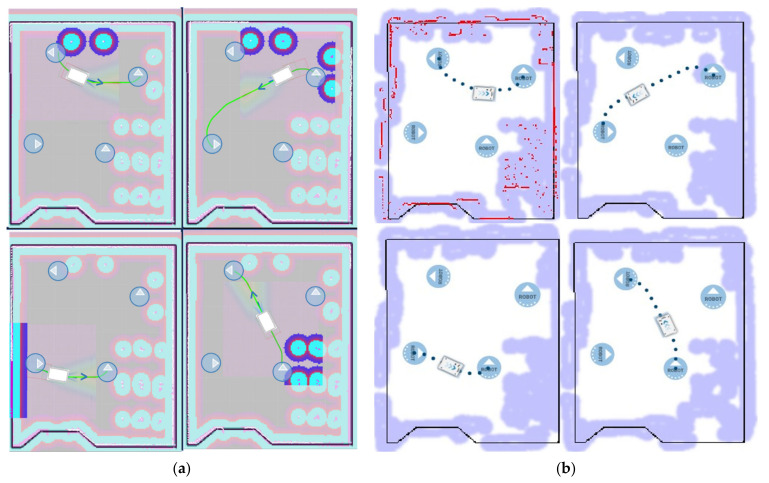
Representation of the mission trajectories in Scenario 1, (**a**) represents the trajectories of the DT; (**b**) represents the trajectories in the real implementation.

The accuracy of the times is also due to the calculation of the trajectories. Figure 8 shows a comparison of the trajectories in DT (a) and in the real world (b), which are practically identical. In this sense, the DT not only performs a prediction of the times but also allows the obtention of the trajectories of the AGV in the industrial plant.

The circles with arrows represent the orientation of the targets. The AGV trajectories are defined with a green line in the simulated version (Figure 8a) and with dots for the real implementation (Figure 8b).

In many trajectories the orientation of the AGV is close to the target because the mobile robot is able to rotate on its own axis. This reduces the distance travelled and the space for maneuvering.

The obstacles of Scenario 1 can be seen in the DT simulation as well as in the real implementation in different shades than those on the ground. In addition, in the first trajectory of the real implementation, the obstacles detected by the AGV’s LIDAR are indicated in red.

Figure 8 shows the trajectories of the Turning-Drilling-Milling mission. The first path goes from the pick and place to the turning process. The next destination is the drilling machine. Afterwards, the AGV moves to the milling machine to finish at the pick and place point.

The results of the actual implementation are obtained from the AGV manufacturer’s application. Therefore, it is guaranteed that the results of the real implementation are independent of the DT. Furthermore, as shown in Figure 8 and Table 4, the results have a high correlation between the real implementation and the DT validating the methodologies and technologies applied in this paper.

### 7.3. Scenario 2

In Scenario 2 the AGV trip times shown in Table 5 are higher than those in Table 4 due to longer distances. In contrast to Scenario 1, in Scenario 2 the maximum speeds are reached due to the larger navigation space. In addition, the accuracy between DT times and the experimental tests is high.

**Figure 9:**
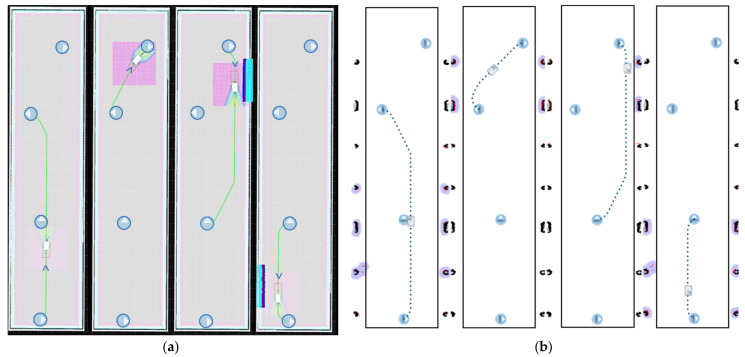
Representation of the mission trajectories in Scenario 2, (**a**) represents the trajectories of the DT; (**b**) represents the trajectories in the real implementation.

As in Scenario 1, the trajectories in Scenario 2 have been evaluated in an analogous way. Figure 9 shows the simulated trajectories of the DT (a) and those of the real implementation (b). In the real implementation the AGV was programmed with a virtual wall represented by a black rectangle.

In this case, the trajectories correspond to the mission “Milling-Drilling-Turning”. For this purpose, the AGV travels from the “pick and place” point through the hypothetical processes of milling, drilling and turning to return to the starting point.

As it can be seen in Figure 9, the paths in Scenario 2 remain almost the same. Thus, it is determined that in large scenarios, both the times and the trajectories are also met.

### 7.4. Navigation and HSI

AGV trajectory calculation is important to identify routes between different targets. Not all routes are valid as there may be traffic, direction and speed restrictions. In this sense, the objective is to obtain the route in the shortest time, which is the main cost of industrial transport. Furthermore, with the DT it is possible to check if this is true for the distribution of the industrial plant.

On the other hand, the HSI facilitates the interaction between the human and the simulation through an interface. In this way, it is not necessary to modify the code each time a mission is established. Furthermore, the HSI runs on the Node-red server which is equipped with hyperconnectivity, so the user would get all the information needed to run the simulation.

**Figure 10:**
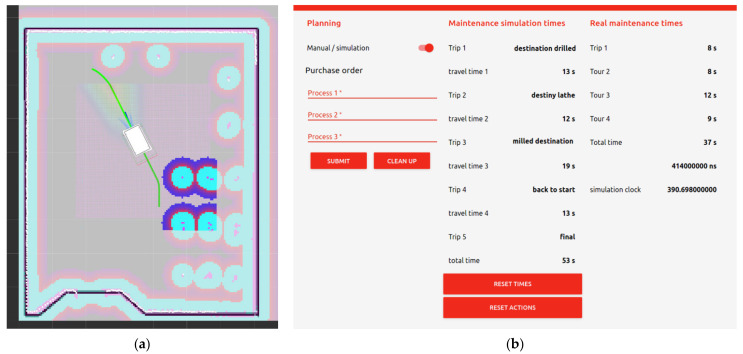
(**a**) Trajectory calculation in the ROS environment in Scenario 1; (**b**) The HSI where mission tasks are assigned and simulation times are obtained.

Figure 10 shows the AGV trajectory calculation in the ROS environment and the HSI for mission assignment.

### 7.5. Discussion

**Figure 11:**
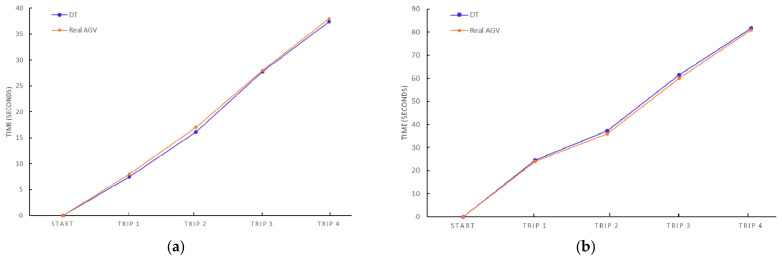
Actual AGV and DT times of trips for a mission (**a**) performed in Scenario 1; (**b**) performed in Scenario 2.

The results shown in the Table 4 and in the Table 5 indicate a very high approximation between the DT and the AGV implemented in reality. Due to the numerous variables (e.g., velocity, accelerations, accurate targets, sensor resolution, trajectory weightings) that affect the navigation and therefore the transport time, it is a positive result. Furthermore, it is the first time that a DT is developed for AGVs. Moreover, it can be seen in the results, the validity of the DT for automated transport with AGVs is demonstrated. In order to visualize the correlation between the operation of the AGV in the virtual and real world, we provide in Figure 11a more detailed definition of the trip of the AGV through the partial times of the missions projected for the satisfaction of the MRP in Scenarios 1 and 2.

Figure 11 shows the minimal deviation between the behaviour of the AGV not only in the general trip and the trajectories followed, as it has been shown in previous subsections, but also in the particularities of each mission in both scenarios. The differences between the partial times are mainly due to potential errors in the relative localization of the AGV in the industrial plant which produces the minimal differences in the calculation of the trajectories and the behaviour of the AGV. Also, sensors actuating in the real world have associated errors in the perception of the scenario inducing errors in the paths followed during the missions.

Besides, the DT provides MES systems with more accurate information about industrial AGV transport without the need for real-world testing. This scientific novelty makes it possible to detect possible problems in planning and thus save costs.

However, the possibilities of this ecosystem allow the automation of both DT simulations as well as industrial plant processes by other external services. For example, the implementation of a problem-solving service (e.g., JSSP, FLP) using AI. Furthermore, the communication architecture can be used to develop the automation of decision making with the industrial plant equipment.

## 8. Conclusions

In the fourth industrial revolution, digitalisation is involved in all processes in order to generate more added value to products and services. In this regard, new technologies (e.g., Cloud Computing, Digital Twin, Industrial Ethernet, CPS) should be used to optimise the performance and efficiency of systems.

The DTs are defined as the virtualisation of the CPS in order to analyse its behaviour under certain hypotheses. In this paper, a DT has been implemented to virtualise automated AGV transport in an industrial plant for providing high-value information for MES systems and organizing the scheduling of the MRP. The implementation of the DT for the AGV navigation presents some challenges (i.e., virtualisation of the environment, numerous variables for AGV modelling, connectivity, programming) addressed in this paper for achieving acceptable results.

The DT proposed acts as an external service integrated in a communications architecture providing the framework for simulations giving critical information for the organization of processes of the Industry 4.0.

In order to validate the DT results, a real communications architecture has been implemented to coordinate the different services and systems located in the industrial plant. This architecture adopts the principles of Industry 4.0, as it uses protocols based on Industrial Ethernet and is protected by cybersecurity. This platform allows the connection with different offsite services through Cloud Computing.

The DT is a server connected to the communications architecture located outside the industrial plant. In this server, a framework of applications and libraries has been created to provide a simulation service which supposes a novelty in the scientific literature of the DTs. Moreover, hyperconnectivity techniques have been used to improve the integration of the human with the virtualisation of the DT.

Furthermore, the demonstrator implemented has the characteristics of Industry 4.0. One feature is to ensure cybersecurity through the use of firewalls and VPN access configurations. Besides, offshoring of services is achieved by using cloud computing techniques. In this way, services are ready for multiple clients from any device connected to the internet. In addition, DT is implemented with the ability to modify the environment at simulation time. The remote services can even communicate with industrial plant equipment via IIoT.

The results obtained in this paper show a correlation of more than 97% between the DT simulation for the AGV trajectories planning and execution time of the missions proposed and the AGV navigation in actual scenarios coordinated through the communications architecture introduced thus validating the DT performance.

The DT ecosystem for industry proposed in this paper is a new vision. Due to the experimentation environment, it lays the foundation for the development of future applications. With the developed simulation environment, more AGVs can be added simultaneously in order to see how they behave in the fleet. Moreover, cobots can be added to simulate pick and place times on manufacturing machines and the combination of both. Furthermore, artificial intelligence-based algorithms for problem-solving such as JSSP or FLP can be hosted in cloud computing due to their high computational power. In addition, direct communication could be established for better optimisation of processes in an automated way.

With all of the above, the coordination and integration of all the technologies associated with Industry 4.0 and SM using the proposed communications architecture is sought. In this sense, the theoretical concepts put forward are validated, fulfilling all the proposed objectives of this paper.

## Figures and Tables

**Table 1:** The coordinates and orientation of the AGV targets are defined for Scenario 1.

Workshop (Machine)	*X*-Axis	*Y*-Axis	Orientation
Drilling	2.6 m	5.3 m	0 degrees
Turning	7 m	7.8 m	90 degrees
Milling	5.5 m	4.8 m	90 degrees
Pick and place	3.5 m	8.8 m	180 degrees

**Table 2:** The coordinates and orientation of the AGV targets are defined for Scenario 2.

Workshop (Machine)	*X*-Axis	*Y*-Axis	Orientation
Drilling	12 m	26 m	0 degrees
Turning	10 m	10 m	270 degrees
Milling	8 m	20 m	180 degrees
Pick and place	12 m	1 m	0 degrees

**Table 3:** Parameters of the AGV missions.

Parameters	Data
AGV model	MIR100 by Mobile Universal Robots
AGV load	0 kg
AGV battery charge	>85%
maximum linear speed	1.5 m/s
maximum angular speed	1.5 rad/s
measurement of times	manufacturer’s application
predefined missions	manufacturer’s software

**Table 4:** Industrial transport times of the missions in the digital twin and in the physical implementation in Scenario 1.

Mission (Targets in Order)	Digital Twin Time (s)	Real Implementation Time (s)	Accuracy (%)
Milling—Drilling—Turning	52.3	51	97.51
Milling—Turning—Drilling	54.1	55	98.36
Turning—Drilling—Milling	55.4	56	98.93
Turning—Milling—Drilling	50.8	50	98.43
Drilling—Turning—Milling	53.1	51	96.05
Drilling—Milling—Turning	37.4	38	98.42

**Table 5:** Industrial transport times of the missions in the digital twin and in the physical implementation in Scenario 2.

Mission (Targets in Order)	Digital Twin Time (s)	Real Implementation Time (s)	Accuracy (%)
Milling—Drilling—Turning	81.6	81	99.26
Milling—Turning—Drilling	95.0	96	98.96
Turning—Drilling—Milling	76.3	75	98.30
Turning—Milling—Drilling	72.5	73	99.32
Drilling—Turning—Milling	95.8	95	99.16
Drilling—Milling—Turning	85.2	87	97.93

## Data Availability

Not applicable.

## Abbreviations

ACK	Acknowledgement
AI	Artificial Intelligence
AR	Augmented Reality
AGV	Automatic Guided Vehicle
CSNA/CD	Carrier Sense Multiple Access with Collision Detection
CSV	Comma-Separated Values
CIM	Computer-Integrated Manufacturing
CPS	Cyber-physical Systems
DPI	Deep Package Inspection
DT	Digital Twin
DCS	Distributed Control System
DDoS	Distributed Denial of Service
ERP	Enterprise Resource Planning
FLP	Facility Layout Problem
HSI	Human Simulation Interface
IE	Industrial Ethernet
IEN	Industrial Ethernet Network
IIoT	Industrial Internet of Things
IoT	Internet of Things
IPv4	Internet Protocol version 4
ISP	Internet Service Provider
JSON	JavaScript Object Notation
JSSP	Job Shop Scheduling Problem
LIDAR	Laser Imaging Detection and Ranging
M2M	Machine to Machine
MES	Manufacturing Execution Systems
MRP	Material Requirement Planning
MQTT	Message Queuing Telemetry Transport
PLC	Program Logic Control
QoS	Quality of Service
RFID	Radio Frequency Identification
ROS	Robot Operating System
SM	Smart Manufacturing
SCADA	Supervisory Control and Data Acquisition
URDF	Unified Robot Description Format
VLAN	Virtual Local Area Networks
VPN	Virtual Network Protocol
VR	Virtual Reality
WAN	Wide Area Network
WLAN	Wireless Local Area Network
